# Are Healthcare Organizations Ready for Change? Comment on "Development and Content Validation of a Transcultural Instrument to Assess Organizational Readiness for Knowledge Translation in Healthcare Organizations: The OR4KT"

**DOI:** 10.15171/ijhpm.2018.95

**Published:** 2018-09-30

**Authors:** Roberto Nuño-Solinís

**Affiliations:** Deusto Business School, Bilbo, Spain.

**Keywords:** Readiness for Change, Measure Development, Implementation, Change Management

## Abstract

Worldwide most health systems are facing a series of common challenges characterized by the increasing burden of chronic diseases and multimorbidity, and the accelerated pace of biomedical and technological innovations, on the other side. There is a growing recognition that many changes are needed at the macro, meso and micro management levels to tackle these challenges. Therefore, knowing if healthcare organizations are ready for change is a key issue, as high organizational readiness for change (ORC) has been positively related with higher organizational effort and staff motivation for overcoming barriers and setbacks in change endeavours. In practice, readiness for change is not commonly measured and there is a need of adequate metrics for it. In this commentary, a new tool for measuring readiness change is reviewed, the OR4KT. It has been developed based on a solid theoretical background and with the involvement of experts and potential users in the design and it has been tested and validated in three languages and in different organizational settings. Although its generalizability needs to be further tested, it seems to be a promising and useful tool to diagnose if organizations are ready to implement evidence-informed changes. A broader recognition of the key role that the science of implementation can play in the success of much needed transformations in healthcare provides a good opportunity for the dissemination of the OR4KT.

## Introduction

Most health systems worldwide are facing a series of common challenges characterized by the increasing burden of chronic diseases and multimorbidity, on the one side, and the accelerated growth of biomedical and technological innovations, on the other side. Together with these factors, other contextual variables also pressure health systems’ key actors, at the micro, meso or macro level, to adopt significant changes, and, in many cases, to embrace system-wide transformations.

Change management is a classical topic of management literature, which has been popular in various sectors including the health sector.^1^ Given the current context where systemic transformations seem to be needed, there is an increasing interest in many international agencies in the implementation of large-scale system transformation as can be seen in many recent reports, eg, the ones of the European Office of the World Health Organization (WHO).^2,3^

Well known authors such as Pettigrew and colleagues^1^ already noted the importance of analyzing receptive contexts for change and they provided a framework which included eight key factors to consider for achieving successful strategic change. These factors are the following: environmental pressure; quality and coherence of the policies; the role of key people leading change; the existence of a supportive organizational culture; managerial and clinical relations; co-operative inter-organizational networks; the fit between the change agenda and the local context; and the clarity of organizational goals and priorities. These factors that are dynamically linked highlight the importance of political, institutional and organizational elements in the receptivity towards change.

Within this context, organizational readiness for change (ORC) is a fertile ground of research and it is a highly needed topic to consider in all major change management projects because there is a proven link between unsuccessful change initiatives and the lack of readiness for change.^4^ Moreover, it has been estimated that around 70% of corporate transformations are unsuccessful^5^ showing that many key success factors are not adequately managed. High ORC is, therefore, a very relevant construct and has been positively related with higher organizational effort and staff motivation for overcoming barriers and setbacks in change endeavours.^6^ This is an important fact to consider not only in large transformational projects but also in the implementation of evidence-based practices contributing to the reduction of the research-practice gap.

Despite the aforementioned evidence and the broad academic consensus on the importance of ORC as a factor that contributes to the success of changes in health organizations and other business sectors, conceptual frameworks, measuring instruments and standardized metrics are rarely used by healthcare managers, with most of the developments related to research purposes or within the proprietary know-how of consultancy firms specialized in change management.

This lack of standardization of tools for measuring ORC contribute to the fact that organizational leaders disregard this concept, and, as stated by Kotter,^7^ half of change management initiatives fail because of the lack of measurement of ORC and, consequently, inappropriate management of the project from the beginning.

Gagnon and colleagues have developed and validated a transcultural instrument to assess Organizational Readiness for Knowledge Translation in Healthcare Organizations, called the OR4KT. The contribution made by these authors is a result of a long term and well documented work that started with theoretical reviews,^8,9^ a review of existing instruments,^10^ a Delphi study and expert panel^11^ that led to the design of a 97-items preliminary version and later to the face validation in three languages of a shorter version, as the research team described in their last paper.^12^ Ultimately, their work led to the development of the OR4KT instrument, a 59 items (grouped under six dimensions) questionnaire, with the purpose of measuring healthcare organizations’ readiness to implement evidence-informed changes.

Although, the initial focus of the tool was on changes related to chronic illness care, the final OR4KT can be applied to help decision makers, healthcare managers and other organizational change leaders to assess change readiness in any kind of interventions provided that a relevant evidence base supported them.

Hereinafter, we briefly comment the existing background information in the literature regarding similar tools and we analyze potentialities, expected benefits and limitations of OR4KT, to finish drawing some conclusions specially designed for potential users.

Starting from seminal models of change as the one of Lewin,^13^ it has been acknowledged the importance of the readiness within the first part of the well-known unfreeze-change-freeze process. In healthcare, the theoretical development of Weiner is the most popular reference.^14^ This author characterized ORC as a multi-level and multifaceted construct that refers to organizational members’ change commitment and change efficacy to implement organizational change. Other authors focused more on structural attributes of the organization, like Armenakis et al^4^ that made a clear distinction between the constructs of organizational and employee readiness for change.

In terms of measurement tools, previous reviews^10,15,16^ identified few valid and reliable ORC measurement instruments. Some examples applied in healthcare settings are the following, the Texas Christian University-ORC (TCU-ORC^17^) instrument that exhibited the best evidence in terms of validity testing; the organizational readiness for implementing change (ORIC) developed following Weiner’s theory by Shea et al,^18^ and, based on Wagner’s Chronic Care Model, Nuño-Solinís et al^19^ developed the Assessment of Readiness for Chronicity in Healthcare Organizations (ARCHO). However, these tools present several limitations that have been discussed elsewhere.^20^ This background justified the big research effort to overcome existing limitations with the development of new tools like the OR4KT that seems promising as it has been designed with the aspiration of being a generic instrument to assess the readiness for change of healthcare organizations related to the implementation of evidence-informed interventions in all kind of healthcare settings. Therefore, the OR4KT operationalizes concepts for the assessment of organizational capacities to engage in any kind of evidence-based change and can be used for evaluating baseline ORC and additionally, it could be used also to monitor changes over a period of time as Grandes et al proposed.^20^

Other strengths of OR4KT are the solid theoretical background and the involvement of experts and potential users in the design. Anyway, the tool needs further validation and application in other contexts with different resource availability and diverse managerial and clinical cultures. In this way, its generalizability can be strengthened.

From a practical point of view, OR4KT allows organizations to quantify readiness for change according to six dimensions: organizational climate for change, contextual factors, change content, leadership/participation, organizational support and motivation. Some of these dimensions are more manageable than others, but provide guidance for organizational leaders, managers, and change agents regarding key aspects that require close attention in change management processes.

However, the tool does not provide a roadmap of the organizational actions needed to increase ORC, nor the implementation strategies that can be adopted in order to increase the success of change efforts. Other relevant practical limitation is that the existing version seems still too long to be implemented in busy practices.

To sum up, OR4KT appears as a promising and useful tool to diagnose if organizations are ready for implementing change. We hope that an increasing practical use and further research can prove higher validity and reliability in different contexts. It is also needed that potential users identify the need to assess ORC in implementation initiatives. To reduce this gap, tools like OR4KT are needed but also a broad recognition of the key role that the science of implementation can play in the success of much needed changes in healthcare.^21-23^

## Ethical issues

Not applicable.

## Competing interests

Author declares that he has no competing interests.

## Author’s contribution

RNS is the single author of the paper.
